# Resveratrol, by Modulating RNA Processing Factor Levels, Can Influence the Alternative Splicing of Pre-mRNAs

**DOI:** 10.1371/journal.pone.0028926

**Published:** 2011-12-13

**Authors:** M. Andrea Markus, Francine Z. Marques, Brian J. Morris

**Affiliations:** Basic and Clinical Genomics Laboratory, School of Medical Sciences and Bosch Institute, The University of Sydney, Sydney, Australia; UMDNJ-New Jersey Medical School, United States of America

## Abstract

Alternative pre-mRNA splicing defects can contribute to, or result from, various diseases, including cancer. Aberrant mRNAs, splicing factors and other RNA processing factors have therefore become targets for new therapeutic interventions. Here we report that the natural polyphenol resveratrol can modulate alternative splicing in a target-specific manner. We transfected minigenes of several alternatively spliceable primary mRNAs into HEK293 cells in the presence or absence of 1, 5, 20 and 50 µM resveratrol and measured exon levels by semi-quantitative PCR after separation by agarose gel electrophoresis. We found that 20 µg/ml and 50 µg/ml of resveratrol affected exon inclusion of SRp20 and SMN2 pre-mRNAs, but not CD44v5 or tau pre-mRNAs. By Western blotting and immunofluorescence we showed that this effect may be due to the ability of resveratrol to change the protein level but not the localization of several RNA processing factors. The processing factors that increased significantly were ASF/SF2, hnRNPA1 and HuR, but resveratrol did not change the levels of RBM4, PTBP1 and U2AF35. By means of siRNA-mediated knockdown we depleted cells of SIRT1, regarded as a major target of resveratrol, and showed that the effect on splicing was not dependent on SIRT1. Our results suggest that resveratrol might be an attractive small molecule to treat diseases in which aberrant splicing has been implicated, and justify more extensive research on the effects of resveratrol on the splicing machinery.

## Introduction

In mammals, alternative splicing is the major means by which cells can generate a diverse repertoire of protein isoforms from a more limited number of genes [1]. It is estimated that 95% of human genes are alternatively spliced [2]. The process of alternative splicing is subject to precise regulation. The utilization of an alternative exon often depends on the cell type, the developmental stage and the influence of intracellular signaling pathways, and this process can occur very rapidly. The importance of correct splicing is apparent from the growing number of human diseases that are recognized as being caused by missplicing [3], [4]. These diseases result from either mutations, as in the case of frontotemporal dementia and Parkinsonism linked to chromosome 17, or deregulation of the cellular splicing machinery, as exemplified by changes in alternative splicing seen in various cancers [5]. Mutations in *cis*-elements are often responsible for aberrant gene expression in various diseases, but tissue-specific splicing factors, by effects on pre-mRNA processing, have also emerged as a cause of pathogenic abnormalities in gene expression [6]. Since different isoforms of proteins can have different physiological effects [7], alternative splicing has emerged recently as a new target for pharmacological intervention [8]. Despite the increasing number of substances, including natural ones such as caffeine, that have been found to be able to modulate alternative splicing [9], [10], [11], toxicity of some and lack of specificity of others remain major caveats.

Resveratrol is a polyphenolic flavonoid found in grape skins and seeds, red wine, blueberries, mulberries, peanuts and rhubarb [12]. Resveratrol affects many major biological processes [13]. In various ways it has been shown to protect against cardiovascular disease, type 2 diabetes and neurological disorders (see reviews: [12], [14], [15]). The first-observed, and now well-established, effect of resveratrol was its ability to inhibit the initiation and growth of tumors in a range of cancer models in mice and rats (reviewed in [12]).

The molecular mechanisms of action of resveratrol may involve intracellular pathways that overlap with ones activated by calorie restriction [16]. It was thought that an early target of resveratrol was the sirtuin class of NAD^+^-dependent deacetylases [17], seven of which (SIRT 1–7) have been identified in mammals. The extent to which the sirtuin-activating actions of resveratrol are direct or indirect is still unresolved, although recent evidence suggests that indirect effects might be more important [18], [19]. The pathways regulated by sirtuins include gluconeogenesis and glycolysis in the liver, fat metabolism, stress resistance and cell survival. Downstream targets of sirtuins are members of the forkhead box O (FOXO) group of transcription factors, as well as p53, PGC-1α, NF-κB, and AGTR1. The result is activation or suppression of genes involved in apoptosis, antioxidant activity, DNA protection and inflammatory pathways [20], [21]. Other direct or indirect targets of resveratrol include kinases such as class IA phosphoinositide 3-kinase and AMP kinase [22], [23]. It remains to be shown whether resveratrol acts by universal, independent or overlapping mechanisms, but its effects are likely to be mediated by a network of genes rather than by linear pathways [24].

It has been found recently that resveratrol can bind directly to DNA and RNA [25]. This led us to ask whether resveratrol might be involved in alternative splicing. As a key process in the regulation of gene expression, alternative splicing is able to influence virtually all cellular processes, including apoptosis, cell cycling, DNA repair and glucose metabolism [26]. In a cell line (fibroblast 3813 cells) used as a model of the neurodegenerative disorder spinal muscular atrophy, resveratrol increased modulated splicing of survival motor neuron-2 (SMN2), promoting exon 7 inclusion [27]; this effect might be relevant to disease treatment. The aim of the present study was to test the hypothesis that resveratrol can modulate alternative splicing of specific primary mRNA transcripts and do so by altering the levels of specific RNA processing factors.

## Methods

### Chemicals

Resveratrol (Sigma) was dissolved in 100% dimethylsulfoxide (DMSO) to give a 1 M solution. This was diluted with Dulbecco's Minimal Essential medium to achieve the final concentrations described in Results. For control experiments resveratrol was omitted and the vehicle only was used. All other chemicals were purchased from Sigma unless stated otherwise.

### Cell culture and transient transfection

HEK293 cells (ATCC, CRL-1573) and HeLa cells (ATCC, CCL-2) were grown in Dulbecco's Minimal Essential medium containing sodium pyruvate (GIBCO Invitrogen), supplemented with 10% fetal bovine serum (FBS; GIBCO Invitrogen) and 5 U/ml penicillin/streptomycin. All cells were maintained at 37°C in an atmosphere of 5% CO_2_. Prior to transient transfections, cells were grown to 50–60% confluency in 6-well plates and then transfected using Lipofectamine 2000 (Invitrogen) according to the manufacturer's instructions. For SIRT1 knockdown, cells were transfected with 100 pmol of *SIRT1*-specific siRNA (Stealth Invitrogen, Cat no. SIRT1-VHS50608) using 4 µl of RNAiMax (Invitrogen).

### Splicing assays

The splicing factors investigated were SMN2, SRp20, CD44v5 and tau. The following minigene plasmids, kindly donated by S. Stamm, University of Kentucky, were used: pSMN2wt (exons 6–8 SMN2 minigene), pXB(x16) (exons 3–5 SRp20 minigene), pETv5 (exon v5 CD44v5 minigene), and pSVIRB-tau-SV9/10L/11 (exons 9–11 tau minigene) [28]. Splicing assays were performed essentially as described [28]. Briefly, 2 µg splicing reporter minigene was transfected into HEK293 or HeLa cells grown on 6-well plates, using Lipofectamin 2000 (Invitrogen) according to the manufacturer's instructions. Twenty four hours after transfection cells were treated with resveratrol (1, 5, 20 and 50 µM final concentration) for 18 h. RNA was then isolated using 1 ml TRIzol (Invitrogen). For the SMN2 minigene, RT-PCR was performed using the primers pcl forward, 5′-ggtgtccactcccagttcaa-3′, and SMNex8rev, 5′-gcctcaccaccgtgctgg-3′, and 30 cycles of 95°C for 30 s, 60°C for 1 min and 72°C for 1 min. For RT-PCR of minigene SRp20, primers were T7, 5′-taatacgactcactataggg-3′, and X16R, 5′-cctggtcgacactctagatttcctttcatttgacc-3′, and 25 cycles of 95°C for 30 s, 55°C for 30 s and 72°C for 1 min. PCR products were then separated in 2% agarose gels. The resultant splicing band pattern (upper band indicating exon inclusion, lower band exon spliced) was quantified using Image J software [29]. Statistical analysis was performed using SPSS and analysis of variance corrected by Dunnett's test for repeated measures.

### Antibodies

Anti-RBM4 (Cat. no 11614-1-AP), anti-PTBP1 (Cat. no 12582-1-AP), anti-hnRNPA1 (Cat. no 11176-1-AP) and anti-U2AF1 (Cat. no 10334-1-AP) were all from Proteintech Group, Inc. Anti-HuR (Cat. no 39-0600) was from Invitrogen. Anti-SF2/ASF for Western blotting was from Invitrogen (Cat. no 32-4500). Anti-SF2/ASF for immunofluorescence was from Santa-Cruz (Cat. no sc-10254). Primary antibodies were used at dilutions of 1∶200 to 1∶3000.

Secondary antibodies were: Alexa Fluor 488 goat anti-rabbit or anti-mouse IgG, Alexa Fluor 594 goat anti-mouse or anti-rabbit IgG, Alexa Fluor 594 donkey anti-goat (all from Invitrogen), as well as sheep anti-mouse horseradish peroxidase (HRP)-linked and donkey anti-rabbit HRP-linked (GE Healthcare). Secondary antibodies were used at 1∶500 to 1∶3000 dilution.

### Western blotting

Whole cell protein extracts were prepared using RIPA buffer (Sigma). Twenty micrograms of extracted proteins were resolved by 12% SDS-PAGE. Proteins were electroblotted on to a PVDF membrane (Millipore). Membranes were blocked for one hour in 5% skim milk, then incubated overnight with primary antibody in blocking solution, followed by 1 h with secondary antibody, before detection by ECL-plus (GE Healthcare) according to the manufacturer's instructions. Images were captured with a Fluorchem HD2 Imager (AlphaInnotech). Bands were quantified using Image J software [29].

### Intracellular localization studies

For visualization of fluorescence, cells were grown on Lab-Tek chamber slides (Nunc) and fixed with ice-cold methanol for 2 min. Following 1 h of blocking with 10% goat serum (Sigma), the cells were incubated sequentially with primary and secondary antibody for 1 h each, then stained with 300 nM DAPI (4′,6-diamidin-2-phenylindol) (Molecular Probes, Invitrogen) and mounted with glycerol/gelatin/PBS (Sigma). Two- and three-channel fluorescent images were acquired on a Zeiss Axioplan 2 imaging microscope with an Axiocam HRm camera.

## Results

### Resveratrol modulates alternative splicing of selected RNA transcripts

To assess the effect of resveratrol on alternative splicing we transfected HEK293 cells with different minigene plasmids which contained alternatively spliceable exons flanked by introns [28]. We then treated the cells with different concentrations of resveratrol (1, 5, 20 and 50 µM), which were well below toxic levels, or with vehicle only (control), and after 18 h of treatment we performed splicing assays. From results of imaging of PCR products on the agarose gels, percentage exon inclusion was calculated as the ratio of signal for the upper band and sum of signals for upper and lower bands.

**Figure 1:**
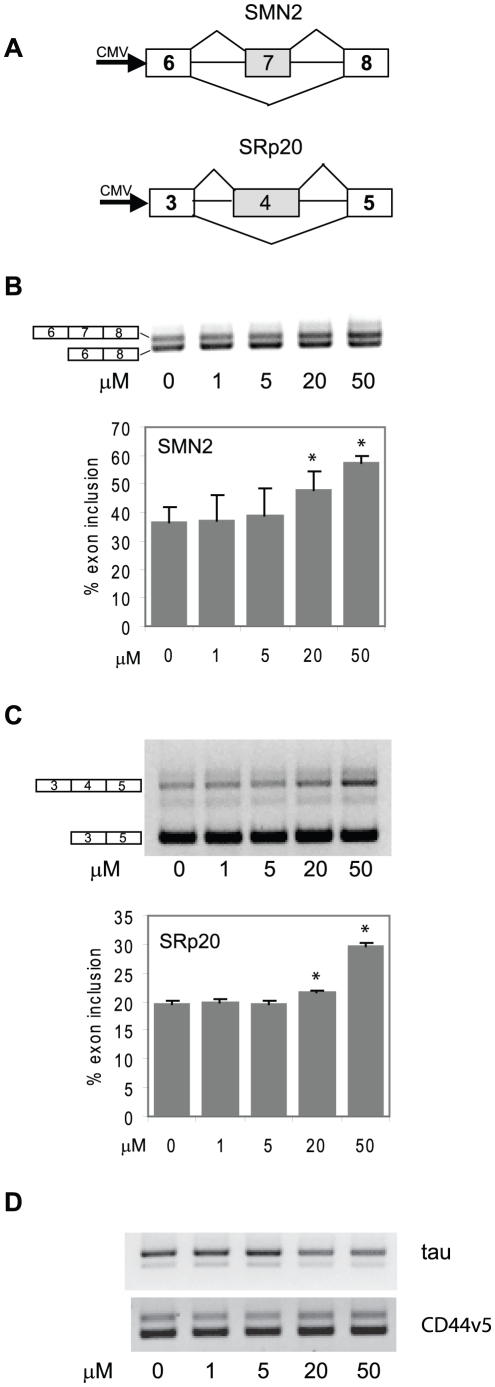
Resveratrol is able to influence alternative splicing of specific RNAs. (A) Schematic of minigenes SMN2 and SRp20. Shaded boxes are alternatively spliceable exons. (B) and (C) Resveratrol caused a significant dose-dependent increase in exon-inclusion for SMN2 (B) and SRp20 (C) RNAs, respectively. Upper panels show representative agarose gels from 3 independent experiments. (D) Representative gels of splicing assays with tau and CD44v5 minigenes, where ratios of upper and lower splicing bands did not change in response to resveratrol. *P<0.05.

This experiment showed that resveratrol was able to modulate alternative splicing in a dose-dependent and RNA-specific manner (Figure 1), while DMSO vehicle alone had no effect on the splicing pattern (data not shown). We saw a strong and significant increase in exon inclusion with SRp20 and SMN2 minigenes (Figure 1A), but no change in splicing was observed with CD44v5 and tau minigenes (seen as no change in ratio of upper and lower bands after resveratrol treatment). This suggests that the effect of resveratrol is specific for certain pre-mRNA targets, but had no effect on others. We were able to confirm the effect of resveratrol on SRp20 and SMN2 minigene pre-mRNA splicing in another cell type (HeLa cells) (data not shown).

### Resveratrol influences the levels of protein of selected splicing factors

**Figure 2:**
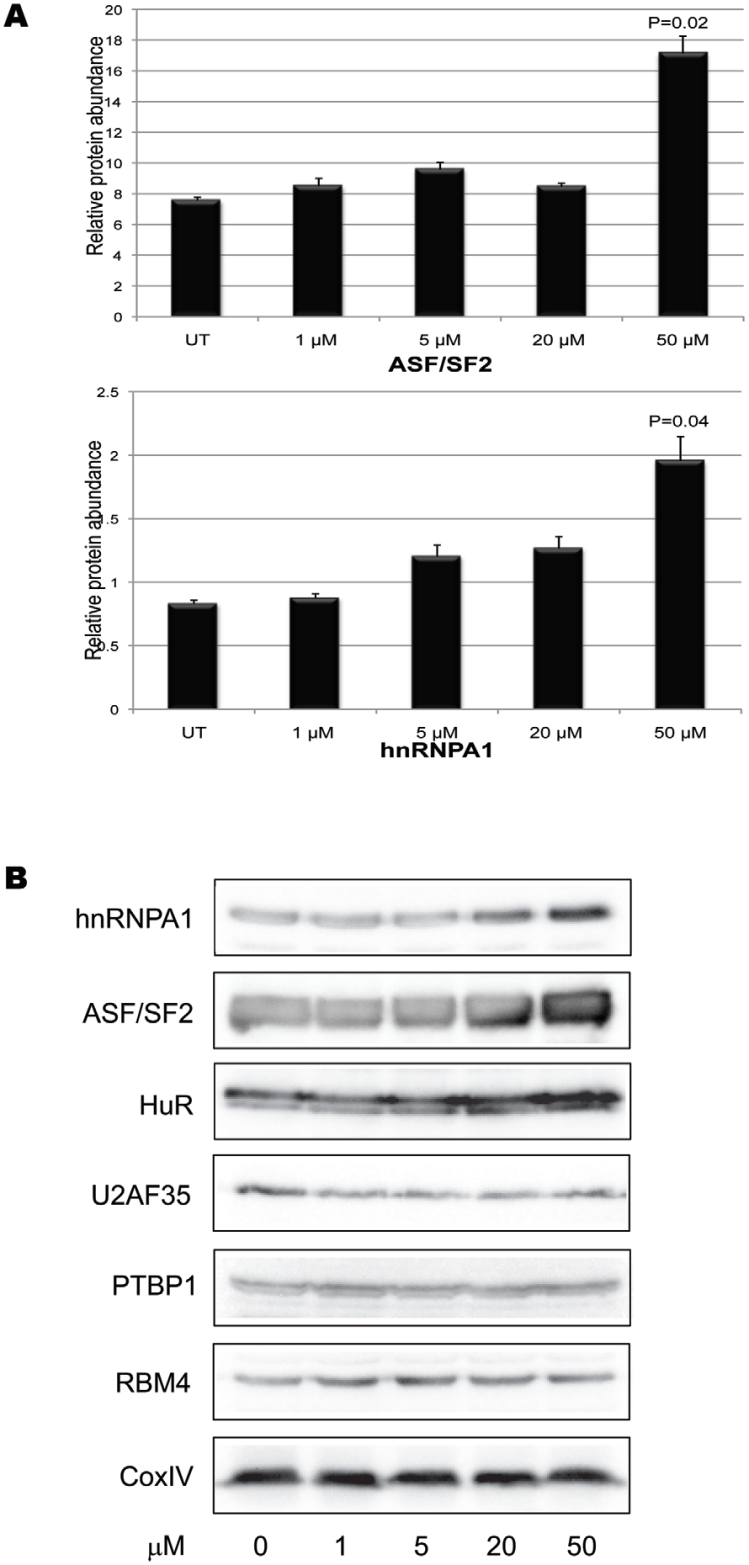
Resveratrol selectively affects specific splicing factor protein levels. (A) Levels of protein of the splicing factors ASF/SF2 and hnRNPA1 in response to treatment with 0, 1, 5, 20 and 50 µM resveratrol. *Significant *P* values (<0.05) are indicated. (B) Representative Western blots showing protein levels of the splicing factors indicated in response to treatment with 0, 1, 5, 20 and 50 µM resveratrol. CoxIV (cytochrome c oxidase subunit IV) was used as a loading control.

We hypothesized that resveratrol might be influencing alternative splicing via an effect on the levels of splicing factor proteins in cells. To test this we treated HEK293 cells with increasing amounts of resveratrol and performed Western blotting with antibodies to a battery of proteins involved in alternative splicing, namely ASF/SF2 [30], hnRNPA1 [31], HuR [32], RBM4 [33], PTBP1 [34] and U2AF35 [35]. We found that resveratrol was able to increase the levels of ASF/SF2, hnRNPA1 and HuR significantly, but did not alter levels of RBM4, PTBP1 and U2AF35 (Figure 2). This suggested that resveratrol is capable of acting selectively so as to alter protein levels of some, but not all, splicing factors.

### The effect of resveratrol on alternative splicing is rapid and independent of SIRT1

**Figure 3:**
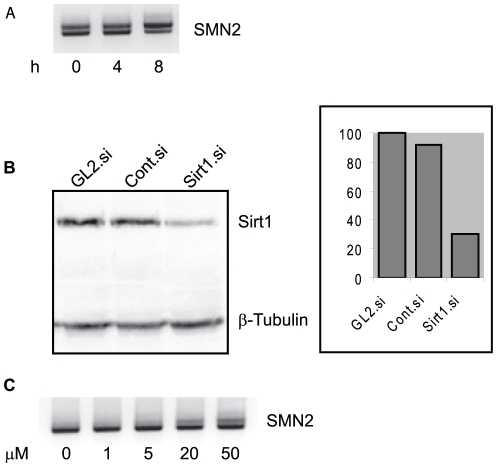
Effect of resveratrol on exon inclusion is rapid and independent of SIRT1. (A) Exon inclusion increases as early as 8 h after exposure of cells to 20 µM resveratrol. Shown is a representative agarose gel of a splicing assay with SMN2. (B) Western blot that shows SIRT1-specific RNAi reduced SIRT1 protein by 70%, whereas control siRNA had no significant effect on SIRT1 level. β-tubulin was used as loading control. (C) Agarose electrophoresis gel following splicing assay for SMN2 after *SIRT1* siRNA and resveratrol treatment at the doses indicated. This experiment showed that the increase in exon inclusion was independent of SIRT1 protein levels.

We then tested the time-course of the action of resveratrol on alternative splicing using the SMN2 minigene. By 8 h of exposure to 20 µM resveratrol an increase in exon inclusion was evident (Figure 3A).

SIRT1 is regarded as a major target of resveratrol [36], albeit by an indirect action, and many of the effects of resveratrol appear to be mediated by activation of SIRT1 [21], [37], which then activates a number of molecular pathways. We therefore tested whether the effect of resveratrol on alternative splicing is dependent on SIRT1. To this end we depleted cells of *SIRT1* mRNA by siRNA-mediated knockdown. As shown in Figure 3B, when using a *SIRT1*-specific siRNA, SIRT1 protein levels were reduced by 70% compared to control siRNAs. Co-transfection of SIRT1-specific siRNA and SMN2 minigene and subsequent splicing assays failed to show an alteration in the effect of resveratrol on alternative splicing of SMN2 (Figure 3C). This suggested that the effect of resveratrol on alternative splicing was independent of changes in SIRT1 activity or gene expression.

### Resveratrol does not alter the intracellular localization of splicing factors

**Figure 4:**
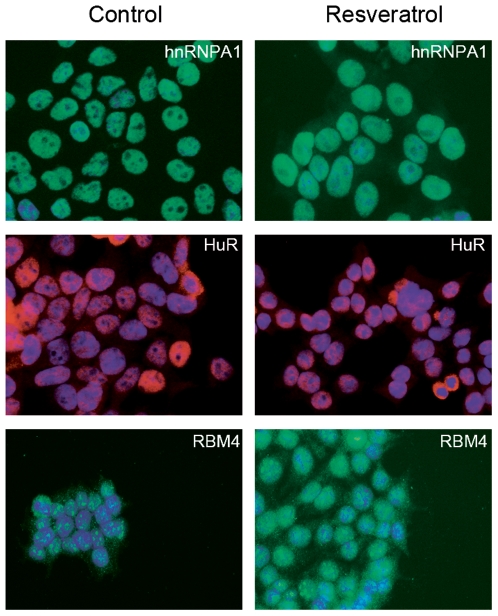
Resveratrol does not influence the intracellular localization of splicing factors. Immunofluorescence results for HEK293 cells treated with 20 µM resveratrol for 18 h. Shown is immunofluorescent signal obtained for the specific splicing factor antibodies indicated. Left panel: untreated control cells. Right panel: resveratrol-treated cells. The nucleus was stained with DAPI. Shown are merged images of DAPI staining (blue) and splicing factor staining (red or green, depending on the secondary antibody used). Scale bar: 20 µm.

Splicing factors often have multiple functions within cells, and their functions can change depending on their localization within the cell. For instance SR proteins such as ASF/SF2 or SRp20 also play an important role in the regulation of translation and do so by relocating from the nucleus to the cytoplasm. We therefore examined whether resveratrol affected the intracellular localization of the splicing factors HuR, hnRNPA, ASF/SF2 and RBM4. Treatment of HEK293 cells with 20 µM resveratrol for 18 h did not alter the localization of any of these splicing factors, each of which remained in the nucleus. Results for HuR, hnRNPA1 and RBM4 are depicted in Figure 4.

## Discussion

The present study has shown that resveratrol can alter the pattern of alternative pre-mRNA splicing. Our study has also demonstrated that this effect may involve an influence of resveratrol on the levels of or activation of specific splicing factors. Resveratrol promoted exon inclusion in an RNA-specific manner in that it changed the alternative splicing of two minigenes we tested (SMN2 and SRp20), but not that of two others. In addition, resveratrol had a selective effect on the levels of splicing factors, being able to increase ASF/SF2, hnRNPA1 and HuR proteins, but not RBM4, PTBP1 and U2AF35. The fact that ASF/SF2 and hnRNPA1 directly influence the splicing of the specific minigenes affected by resveratrol, but not that of the minigenes whose splicing was not affected by resveratrol, supports our findings and points, at least in part, to the mechanisms involved. In particular, it was shown earlier that ASF/SF2 and hnRNPA1 are both involved in exon 7 splicing of SMN2 [38] and that ASF/SF2 is involved in exon 4 splicing of SRp20 [39]. Thus resveratrol has effects that are specific for some, but not all, splicing events.

Given the importance of alternative splicing in various diseases, the identification of non-toxic compounds that inhibit certain splicing events or stages in the splicing reaction specifically is of considerable potential importance. The use of RNA-binding molecules as antibiotics (e.g., gentamicin or tetracycline) illustrates that drugs can work by targeting RNA and/or RNA-binding proteins [40]. Furthermore, several low-molecular-mass compounds, such as histone deacetylase inhibitors or kinase/phosphatase inhibitors, have been identified as modulators of alternative splicing [11]. The low degree of specificity that these have on numerous splicing events has to date been problematic however. Currently, only the use of oligonucleotides allows the unambiguous, specific targeting of a particular splicing event. The potential specificity of resveratrol towards a limited repertoire of splicing factors is therefore of interest and would justify its examination on a larger scale. Splicing factors, such as ASF/SF2 that are key protein constituents of the core splicing machinery, are master-regulators of gene expression post-transcriptionally, and are involved in diverse processes, including mRNA nuclear export, nonsense-mediated mRNA decay and translation [41]. Our data showing an effect of resveratrol on specific splicing factors could suggest that such actions might impact on all of the above, and in so doing may be responsible in part for the wide range of beneficial effects that resveratrol has on, for example, the cardiovascular system, cell growth and metabolism [37].

Our findings are in line with the ability of other botanical compounds, such as epigallocatechin gallate and curcumin to induce alternative splicing of full-length *SMN2* [27] and of resveratrol to inhibit tumour necrosis factor-alpha (*TNF*-α) pre-mRNA splicing in fish [42]. Our findings in human cells add impetus to the need for extension of testing of resveratrol on alternative splicing in mammalian cell lines and cells of other species.

The effect of resveratrol on pre-mRNA splicing factors shown here could contribute in large part to the influence that resveratrol is known to have on phosphorylation of target of rapamycin (mTOR) [43]. mTOR activates S6K1, which phosphorylates eIF4B and S6, so promoting translation initiation, and this may be enhanced by ASF/SF2 [44].

In what way resveratrol is able to affect splicing factor expression is yet to be determined, but could involve its ability to activate transcription factors such as FOXOs [45]. Our siRNA-mediated *SIRT1* knockdown experiments show, moreover, that the effect of resveratrol on splicing and splicing factor levels is not dependent on SIRT1 expression or activity. Thus SIRT1 deacetylase actions appear not be involved in the effect that resveratrol has on splicing.

In conclusion, we have found that resveratrol is a specific modulator of alternative splicing. As such it could be of value in treatment of diseases caused by aberrant splicing. Larger scale studies and experiments in animal models of diseases will be needed to support the clinical utility of this effect of resveratrol.
